# Raising Awareness about Continuing Health Education with Nursing Professionals: Strategic Action Research

**DOI:** 10.1590/0034-7167-2025-0225

**Published:** 2025-12-08

**Authors:** Cloves Roberto Felden da Silva, Juliana Silveira Colomé, Oclaris Lopes Munhoz, Liliane Alves Pereira, Francine Casarin, Silomar Ilha

**Affiliations:** I Universidade Franciscana. Santa Maria, Rio Grande do Sul, Brazil; II Universidade Federal de Santa Maria. Palmeira das Missões, Rio Grande do Sul, Brazil; III Faculdade Integrada de Santa Maria. Santa Maria, Rio Grande do Sul, Brazil

**Keywords:** Education, Continuing, Knowledge, Hospitals, Qualitative Research, Nursing., Educación Continua, Conocimiento, Hospitales, Investigación Cualitativa, Enfermería.

## Abstract

**Objectives:**

to analyze the contributions of an awareness-raising workshop to nursing professionals’ knowledge about continuing health education.

**Methods:**

strategic action research, developed with 25 nursing professionals from a private hospital, located in Rio Grande do Sul, Brazil. Data were collected between January and August 2024, using a questionnaire with open questions, before and after an awareness-raising workshop. A data discursive textual analysis was performed.

**Results:**

the central category “Understanding about continuing health education” emerged, unitized in five categories of analysis that dealt with the (lack of) knowledge about continuing health education and the Continuing Health Education Center, a scenario that changed after awareness raising.

**Conclusions:**

it was revealed that there was a lack of knowledge or superficiality about the topic before the awareness-raising sessions. Afterwards, it was possible to identify the expansion of knowledge, both in its conceptual and functional aspects. Awareness-raising workshops contribute to transformation of knowledge.

## RESUMEN

**Objetivos:**

analizar las contribuciones de un taller de sensibilización al conocimiento de los profesionales de enfermería sobre educación continua en salud.

**Métodos:**

investigación-acción estratégica, desarrollada con 25 profesionales de enfermería de un hospital privado ubicado en Rio Grande do Sul, Brasil. Los datos se recopilaron entre enero y agosto de 2024 mediante un cuestionario con preguntas abiertas, antes y después del taller de sensibilización. Se realizó un análisis textual discursivo de los datos.

**Resultados:**

emergió la categoría central “Comprensión sobre educación continua en salud”, unificada en cinco categorías de análisis que abordaron el (des)conocimiento sobre educación continua en salud y el Núcleo de Educación Permanente en Salud, escenario que se transformó a partir de la sensibilización.

**Conclusiones:**

se reveló que existía desconocimiento o superficialidad sobre el tema antes de las sesiones de sensibilización. Posteriormente, se observó una ampliación del conocimiento, tanto en sus aspectos conceptuales como funcionales. Los talleres de sensibilización contribuyen a la transformación del conocimiento.

## INTRODUCTION

The hospital context of healthcare is one of the most important areas for healthcare professionals’ work, especially those who make up the nursing team, since these are the professionals in greatest number in this setting, in addition those who spend most of their time in direct contact with hospital routines and care. In this context, work processes are necessary that allow them to exercise action-reflection-action in this setting of action that, at times, presents rigid, pre-established and verticalized routines. Thus, continuing health education (CHE) stands out, with a view to quality of care, because with the evolution of work processes in Brazil and in the world, it has become essential to think of strategies that allow for the production of harmony between the health field needs and the effective response capacity of all professionals^(1)^.

CHE is committed to a transformative approach to healthcare practices, based on territorial and socioeconomic specificities^(1)^. Therefore, knowing the potential of CHE in healthcare services is an essential element to overcome adversities and highlight its potential^(2)^. Through CHE, professionals learn/strengthen the most recent and evidence-based practices, which enables/raises awareness to provide care that promotes patients’ quality of care and safety, including the use of new tools, technologies and behaviors that can improve health outcomes^(3)^.

In this regard, aware of the need for CHE actions, healthcare institutions from different regions of Brazil have invested efforts in the implementation of Centers for Continuing Health Education (In Portuguese, *Núcleos de Educação Permanente em Saúde* - NEPS)^(4,5)^. NEPS are characterized as a strategic management proposal, with a view to consolidating an organized service to plan and execute actions to qualify health practices, through training processes, with varied methodologies together with healthcare professionals. The implementation of NEPS requires intersectoral work capable of articulating individual and institutional development^(6)^.

Although the implementation of NEPS is not included as a guideline in the Brazilian National Policy for Continuing Health Education (In Portuguese, *Política Nacional de Educação Permanente em Saúde* - PNEPS)^(7)^ and in Ordinance GM/MS 1,996/2007, which provides guidelines for the implementation of PNEPS^(8)^, it has proven to be an important tool for consolidating CHE in different settings. In this regard, research conducted in the state of Paraíba demonstrated that the implementation of NEPS contributed to collaborative work, which enhanced the feasibility of actions to strengthen CHE. In addition, the research showed that NEPS contributed to coordinators’ interest in appropriating different approaches to active work methodologies with staff^(4)^. Another study carried out in the state of Maranhão demonstrated that the implementation of NEPS was fundamental, due to its transformative nature, improvement in quality of care and qualified, responsible and conscious care promotion^(9)^.

NEPS are intended to elaborate, plan, support and execute in an articulated manner proposals that involve educational actions in the workplace so that learning and teaching are incorporated into the daily routine of services and health work^(10)^. Thus, in institutions that have implemented NEPS, CHE activities are strategies developed mainly by its actions, with the aim of improving worker training processes and reorganizing health practices, based on a theoretical framework of teaching and learning^(4-9)^.

However, although the relevance and need for NEPS in healthcare services is understood, it is still possible to see that it is not a reality in all institutions, as in the setting of this research, which requires attention and investment for its implementation, maintenance or improvement of healthcare service quality. Thus, it becomes necessary to understand what healthcare professionals in these institutions know about CHE and about NEPS as a tool to enhance actions and CHE, with a view to situational diagnosis that makes it possible to think of strategies for its future implementation^(11)^.

Furthermore, it is understood that in order to implement a work proposal that enables the implementation of a NEPS in the research setting, it is essential that professionals understand the importance of CHE and NEPS as a tool to enhance educational/training actions. To this end, it is crucial to invest in methodologies and strategies that contribute to professionals’ knowledge on the subject. In developed countries, investment in activities that provide training for healthcare professionals has been considered a widely used intervention strategy^(12)^.

Among the possible methodologies for this purpose, awareness-raising/training workshops stand out, as they provide the opportunity to provide participants with the necessary tools, through methodologies that value participatory construction, exchange of experiences and knowledge^(13)^. In a survey conducted with professionals from a hospital in the state of Pará, managers reported the challenges in the CHE process, highlighting the need to dedicate efforts to raising awareness among professionals. Likewise, healthcare professionals recognize the importance and need for different methodologies for raising awareness/training, namely the use of workshops^(11)^.

However, in addition to carrying out intervention actions, it is necessary to assess their contribution/effectiveness, through knowledge production and/or deepening, a fact that justifies the need and relevance of this research that, in addition to offering awareness/training workshops, aims to compare professionals’ knowledge before and after them, which can contribute to hospital management for the implementation of the process of continuous quality improvement. In addition to what has been described so far, it is worth noting that discussions and research on the topic at hand are essential and necessary, since it is highlighted by the National Agenda of Health Research Priorities in the country, more specifically in axis 8 (work management and health education)^(14)^. It is also aligned with objective 3 (“Good health and well-being”) and objective 8 (“Decent work and economic growth”) for sustainable development, presented in the United Nations 2030 Agenda^(15)^. Considering the above, the question is: what are the contributions of an awareness-raising workshop to nursing professionals’ knowledge about CHE?

## OBJECTIVES

To analyze the contributions of an awareness-raising workshop to nursing professionals’ knowledge about CHE.

## METHODS

### Ethical aspects

This research is part of a macroproject called “Implementation of a Center for Continuing Health Education in a private hospital: strategic action research”. The general objective of the project was to build NEPS in a private hospital. This article addresses one of the specific objectives of the project.

This study was submitted to a Research Ethics Committee (REC) via *Plataforma Brasil*. Only after REC approval was the first contact made with participants. The ethical and legal precepts involving research with human beings were considered, in accordance with Resolution 466/12 of the Ministry of Health^(16)^. The Informed Consent Form was signed by participants in two copies, one for the participant and the other for the researchers. Participant anonymity was maintained, identifying them by the letters “N” (nurse) and “NT” (nursing technician), followed by a number (N1, N2...; NT1, NT2...).

### Study design

This is strategic action research^(17)^, the transformation of which was previously planned by the researcher, who was responsible for monitoring the effects and assessing the results of its application. To this end, four phases were followed to conduct this strategic action research, divided into eight stages, as can be seen in Chart 1.

**Chart 1:** Schematic representation of the phases of strategic action research

ACTION RESEARCH		
**Phase**	**Research stages**	
Diagnostic	Exploratory	Identifying the problem within a context Collecting relevant data
	Analytical	Analysis of collected data Meaning of the data collected Identifying change needs
Planning	Finding possible solutions	
Implementation	Intervention action	
Assessment	Transformation	

*Source: from^(18)^.*

To guide the clarity and writing of this report, the COnsolidated criteria for REporting Qualitative research checklist was used^(19)^.

### Methodological procedures

#### Study setting

This research was conducted at a private hospital located in the central region of the state of Rio Grande do Sul, Brazil. The institution was founded in 1976. It currently has 68 inpatient beds, including clinical, surgical, psychiatric and post-cesarean inpatient units. It also has five operating rooms, ten beds in the recovery room and five beds in the Intensive Care Unit, with the purpose of providing adequate care, maintained with resources from its private work and health insurance plans.

The institution has 200 professionals, including 25 nurses, 100 nursing technicians, one nutritionist, four physiotherapists, one psychologist, one administrator, 22 professionals responsible for cleaning, cooks, general service professionals, as well as general practitioners and surgeons.

#### Study participants

Although it is understood that CHE operates through interprofessional logic and that, in a healthcare service setting, everyone cares, for this research it was decided to work initially with the institution’s nursing care team professionals, as they are the ones who work directly in direct care for patients, from the moment of hospitalization until discharge. Furthermore, they are the ones who normally manage (nurses) and organize (nursing team) healthcare services and guide other professionals about updates to institutional standards and routines.

Nurses or nursing technicians who had been working at the institution for at least three months were included, as it is understood that these professionals have already completed their probationary period and are part of the institution’s permanent staff. Professionals who were on medical leave, leave or any other absence during the data collection period were excluded from the study. Twenty-five professionals met the criteria and agreed to participate in the study.

#### Data collection and organization

Data were collected from the 25 participants in two stages. Initially, from January to March 2024, they were individually invited by the main researcher to participate in the research. After acceptance, a semi-structured interview (stage 1) was conducted between June and July 2024, specifically designed for this research, and conducted in a single stage with each participant. The interview script consisted of two parts: the first, with a description of participants, and the second, with the following open-ended questions: what do you understand by CHE? What do you understand by a NEPS?

Data collection was conducted by one of the researchers, a male nurse with experience in hospital settings, who was previously trained to collect data. A pilot test of the instrument was carried out, with the application of interviews with a nurse and a nursing technician who were not part of the sample for this research. The interviews (moment 1) allowed us to identify knowledge gaps on the topic, which led to the need for intervention. Thus, the researchers sought to find possible solutions and, as an intervention, planned workshops to raise awareness among nursing professionals about the importance of CHE and NEPS, which took place in July and August 2024.

Three workshops were held in the hospital units, each lasting an average of 40 minutes. The activity was coordinated by the researcher, a male nurse with experience in the topics addressed. The workshops began by welcoming the participants and thanking them for participating in the research. A PowerPoint presentation with aspects related to CHE and NEPS was used as a visual resource. Professionals were encouraged to contribute to the workshops by sharing their knowledge and experiences. After this initial moment, a collective discussion on the topics was held, providing a means to broaden and deepen knowledge on the aforementioned topics.

After the awareness-raising workshops, from August to September 2024, semi-structured interviews (moment 2) were conducted again with the 25 participants using the same instrument used in the first moment, with an average duration of 40 minutes, with a view to assessing knowledge about the topic after the intervention. It should be noted that all interviews were, with participants’ authorization, recorded manually. They were then transcribed in full, mechanically, by the researchers, with the help of Microsoft Word^®^ (version 16.31). These were returned to participants so that they could validate the information.

#### Data analysis

**Figure 1:**
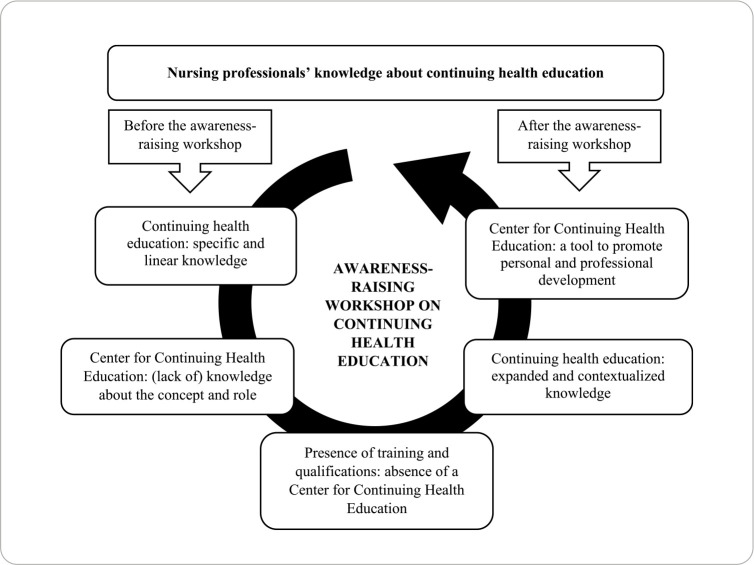
Representative diagram of the interconnection between the central category and the analysis categories

The data were analyzed based on the discursive textual analysis technique, based on its three components: unitarization; the establishment of relationships; and communication^(20)^. Thus, the researchers began by reading the texts intensively and in depth, which led to a central category regarding professionals’ understanding of CHE. Afterwards, the reports initially included in this category were read again in detail, being separated into different units of meaning, from which five categories originated (Figure 1). Finally, the researchers carried out the communication process, at which point they produced the descriptions and interpretations of the phenomena investigated^(20)^.

## RESULTS

Of the 25 participants in the study, 21 were women and four were men, aged between 24 and 53. As for their education, 15 were nursing technicians and nine were nurses. The time since graduation ranged from two to 22 years. Their professional experience ranged from eight months to 21 years, and their experience in the hospital where the study was conducted ranged from eight months to 16 years.

The analyzed data resulted in a central category entitled “Understanding about continuing health education” and in five categories of analysis, as shown in Figure 1.

### Continuing health education: specific and linear knowledge

Before the awareness-raising workshops, nursing technicians had limited and linear knowledge on the subject, since their speeches only dealt with qualifications and training, which does not fully characterize the concept of CHE. Nurses, although they demonstrated a better understanding of the subject, presented speeches that still did not contemplate the definition and objectives of CHE.

*For me, it means learning through training.* (NT2)

*I work with the objective of continuing care focused on users.* (NT4)

*I believe that it involves training and qualifications to maintain professionals’ learning in their work routine.* (NT5)

*Keeping professionals up to date within an institution.* (NT10)

*Strategies for changes in healthcare.* (NT13)

*Monthly or biweekly training in units.* (NT14)

[...] *continuing education on a specific topic in our service.* (N1)

*It has an educational purpose, responsible for professional training.* (N2)

*It includes training to be developed for professionals working in a specific service.* (N4)

*These are actions within the service that seek to qualify professional practice.* (N5)

*Creating educational actions with some topic of problematization in the work.* (N7)

*It is an articulation of practices observing the problems and weaknesses of the sectors and thus transforming these needs into training.* (N9)

### Center for Continuing Health Education: (lack of) knowledge about the concept and role

In relation to professionals’ knowledge about NEPS, before the awareness-raising workshops, some participants referred to CHE. Other professionals referred to NEPS as a sector for conducting training and qualifications.

*It would be a continuing education for healthcare professionals.* (NT1)

*A sector that provides training for staff.* (NT3)

*I believe that the aim is to provide more effective care.* (NT6)

*It would be a sector with someone to provide training for staff.* (NT7)

*I think it would be a sector that provides training.* (NT9)

*I think it serves to introduce new work techniques.* (NT11)

*It serves to improve staff’ professional training.* (NT15)

*Professional qualification and training.* (N3)

*I understand it as a sector responsible for training the teams working in a hospital.* (N6)

Regarding NEPS role, before the awareness-raising workshops, participants mentioned improving services, work techniques, training and continuing education (CE).

### Presence of training and qualifications: absence of a Center for Continuing Health Education

According to participants’ reports, it is possible to see that there are training courses in some sectors of the hospital, which are passed on by the nurses of the units to the teams. However, the research participants were unaware of the presence of a NEPS in the institution.

*No, because we only have training courses that most of the time are already known to professionals, but are often not carried out frequently.* (NT8)

*There is a lack of someone to embrace the cause to be done. There is a lack of people to integrate the group that would aim at improvements, perhaps for this reason it does not yet exist. There is also a high turnover of nurses here at the hospital.* (NT9)

*We still do not have a NEPS; we only have training provided by nurses in the units. The institution lacks organization and planning.* (NT10)

*No, due to weaknesses in development, there is a lack of people to integrate a group. There is a high turnover of nurses here at the hospital.* (NT12)

*No, I believe that because they do not know its real importance and lack of planning* [...] *because investment is needed to hire a responsible professional to head this group, perhaps someone is lacking initiative, I do not know.* (N3)

*No, it does not exist due to a lack of organization and creation, because, in my opinion, there is a lack of interest of administrators and management.* (N5)

*No, I honestly do not know why it does not exist yet; I believe that the turnover of professionals prevents the creation of the continuing education group.* (N8)

*No, the group does not exist, but there are trainings within the institution.* (N9)

In their perceptions, there is a lack of qualified, organized, dedicated professionals with adequate planning who are available to integrate and take responsibility. Professionals attribute the failure to implement NEPS, among other factors, to the lack of organization, investment, professionals to lead the services and the high turnover of professionals at the institution.

### Continuing health education: expanded and contextualized knowledge

After the awareness-raising workshop, improvements were noted in professionals’ knowledge about CHE. Participants demonstrated a broader understanding of CHE’s contribution as a driver of knowledge.

*These are actions that improve work-related knowledge, sharing knowledge through moments of listening and speaking, addressing issues that encourage health promotion and prevention through educational interventions.* (NT2)

*This is any educational activity that aims to problematize the care provided, in addition to serving as a driver of knowledge through acquired and shared experiences. It is a tool for reflecting and sharing experiences, aiming at more qualified work, promoting critical, reflective, committed and technically qualified thinking and delving into certain issues that aim to meet the demands of teams, improving the quality of service.* (NT5)

*It consists of educational actions and refers to individuals’ and populations’ health needs. It delves into certain issues that arise from demands of teams. It aims to improve the quality of services. It is a tool for reflecting and sharing experiences, aiming at more qualified work, transforming the work environment. These are actions designed together with the teams, listening to professionals and understanding their needs and, thus, creating conditions for the qualification of these professionals and improving patient care.* (NT10)

*These are educational actions based on the problematization of the work process in health. A tool for reflecting and sharing experiences, aiming at qualified work, transforming the work environment, promoting critical and reflective thinking. Learning and teaching must be incorporated into the daily routine of organizations and work.* (N4)

*I understand it as a way of observing and asking about professionals’ needs and promoting various actions, seeking to improve work quality and effectiveness.* (N5)

*It is when daily practices are brought in and a teaching, a learning method is organized, where everyone in the healthcare institution can learn and thus improve their work acts, thus transforming these acts in a more effective, qualified and safe manner.* (N9)

As can be seen, after the awareness-raising workshops, the research participants began to understand CHE as a tool that enables moments of listening and dialogue, with opportunities for learning, building knowledge as a professional and improving the quality of service. In addition, the following reports demonstrate participants’ understanding of CHE roles.

*It promotes critical, reflective, committed and technically qualified thinking, and delves deeper into certain topics that aim to meet the demands of teams, improving the quality of service.* (NT4)

*It aims to improve healthcare services, as well as sharing knowledge through moments of listening and speaking, where we have the opportunity to learn, unlearn and rebuild ourselves as professionals.* (NT13)

*In my opinion, continuing education provides opportunities for learning from everyday life, sharing knowledge, moments of listening, learning, encouraging health promotion and prevention of diseases through educational activities.* (NT14)

*It serves to qualify workers, update routines and the constant search for knowledge, and encourages health promotion and prevention through educational interventions with knowledge sharing and moments of listening and speaking.* (N2)

*Continuing education aims to transform knowledge that is reflected in the service, improvement, updating of services, aiming at quality in the expansion of knowledge. Therefore, I understand it as a tool for reflecting and sharing experiences, aiming at quality in and of work* [...]. (N1)

*Learning at work, where learning and teaching are associated with everyday life. Its purpose is to identify professionals’ and service’ needs and, with this, seek the possibility of transforming and improving the performance of health workers.* (N7)

### Center for Continuing Health Education: a tool to promote personal and professional development

After the workshops, it was clear that professionals began to understand NEPS as a driver of personal and professional growth, in which learning and the educational process go hand in hand, improving new work techniques and other needs that arise in daily work.

*A group formed by qualified individuals with the objective of providing the propulsion of knowledge, personal and professional development of professionals.* (NT1)

*Its objective is the staff’ personal and professional development through new knowledge, techniques and work processes, enabling them to provide better care and collaboration with the team.* (NT3)

*NEPS contributes to professionals’ personal and professional development; its objective is to present new knowledge to improve work and the quality of healthcare services.* (NT6)

*Its objective is to provide the personal and professional development of staff, through new knowledge, techniques and work processes.* (NT7)

*A group of professionals responsible for developing the technical, personal and professional growth of staff of a given institution.* (NT9)

*It would be a group of professionals responsible for developing the technical, personal and professional growth of staff of a given institution.* (NT11)

*Providing personal and professional development to staff, through knowledge, equipping them for care in a multidisciplinary team.* (NT15)

*It works to train the multidisciplinary team to improve the quality of services through new knowledge, techniques and work processes, enabling them to adequately perform better healthcare and work with the multidisciplinary team, ensuring quality care.* (N3)

*It is a group formed by people who seek to promote the personal and professional development of staff, through training so that care is adequate with appropriate and safe work techniques and processes for the reality.* (N6)

## DISCUSSION

In this research, it was evident that, before the awareness-raising workshop, some of the participants, especially nursing technicians, were unaware of CHE, since their statements were predominantly about elements such as qualifications and training, which does not reflect the concept breadth and CHE role. Similar data was evidenced in research carried out in a large hospital in the city of Curitiba, in the state of Paraná, where participants referred to CHE as a synonym for CE, not having an in-depth view of the topic and the distinction between the concepts^(21)^.

In this regard, it is worth mentioning that CHE aims at more than just providing training and education, as it represents a culture of education in the service that drives the personal and professional development of the people involved, in which observation, dialogue and active listening must be constantly present so that the needs of the people involved can be identified. It is understood that, from this, it is possible to think and plan actions that contribute to reality, enabling self-reflection and self-assessment, helping each professional to feel like the author/leading star in the journey of their lifelong learning^(22)^.

Thus, it is clear that CHE is a pedagogical concept that should be defined as learning in the workplace, where teaching and learning are formed in the daily routine of organizations and at work. It is linked to significant learning and the possibility of transforming professional practices and the work environment by encompassing various foundations between the reality and experience of each person, requiring the finding of a solution to the highlighted problem. A study developed in China highlighted the importance of CE programs adapted to specific needs to improve nurses’ professional competency^(23)^. Thus, it can be inferred that CHE is characterized as a tool that provides professionals with a proximity between theory and practice, allowing solutions and changes in the setting and assistance provided to the community^(24)^.

Regarding knowledge about NEPS, before the awareness-raising workshops, the professionals participating in the research understood it as a sector for training and qualification of new techniques as well as CE. This data supports those of research developed with the objective of implementing NEPS in a psychiatric hospital in the state of Piauí, which showed similar data, since it was assigned the roles of correcting weaknesses and improving workers’ practice^(25)^.

As can be seen, the participants in this research have a limited and simplistic understanding of NEPS in their statements, attributing to it only qualification and training role. Thus, it is important to mention that planning and carrying out qualification and training in the service are some of the roles of NEPS; however, its relevance in services is not restricted to the execution of specific tasks. NEPS is the structure responsible for working with the reality of services, defining a connection between education and work, in order to resume the CHE process for the development of services and generating an impact on user health^(26)^.

Hence, it is worth mentioning that the lack of knowledge of the conceptual and operational issues of CHE and NEPS, as well as between CHE and CE, provokes reflection on the need to broaden the discussion to academic and technical training, which did not allow the professionals participating in this research to know PNEPS. In addition to this, another relevant factor to be questioned is the lack of spaces for problematization in the service’s daily life.

Based on the reports, it was possible to identify that there were training courses in some sectors of the hospital where this research was conducted, which were provided by nurses in the units. However, there was no NEPS implemented in the institution due to, according to participants, the lack of organization, investment, professionals to lead services and the high turnover of professionals in the institution. Similar data was evidenced in a study carried out in a public hospital, with the objective of understanding the challenges faced by CHE to achieve improvements in quality and patient safety. In the study in question, the challenges covered human issues, such as resistance to change, management issues and high turnover of professionals at the institution^(11)^.

In this regard, research conducted at a hospital in the city of Belém, state of Pará, highlighted the importance of CHE for improving quality. CHE was identified as the basis of this process, where professionals are constantly updated on patient safety protocols and the execution of appropriate procedures to be carried out, encouraging the multidisciplinary team to acquire knowledge, skills and attitudes that contribute to risk management, quality assurance and safety in care processes^(27)^.

Thus, it is worth mentioning that NEPS in healthcare services is a tool that allows professionals already included in the service to constantly update their knowledge and integrate disciplines into different health knowledge. Furthermore, it has routines and schedules for meetings with management representatives, being responsible for assessing health unit workers’ learning needs and developing health education actions^(28)^.

In view of this, CHE actions are opportunities for producing knowledge and greater security, contributing to work processes among professionals, thus strengthening teamwork, since the act of planning requires knowledge of the reality that allows for the establishment of objectives and goals. Therefore, it is important that the NEPS team works together with the institution’s organizational system to ensure its performance despite existing changes^(29)^.

Hence, the need to overcome resistance is highlighted by raising awareness among healthcare professionals about the importance of educational processes^(27)^. In this study, after the awareness-raising workshops, professionals began to better understand the concepts and attributions/contributions of CHE as a driver of technical, personal and professional growth of workers, which has repercussions on quality, efficient and safe care for patients. The same occurred in relation to NEPS, since professionals began to understand it beyond a sector, as a tool that provides the opportunity to put CHE and its benefits into practice, through listening, dialogue, learning and knowledge construction, with repercussions on the quality of service.

Thus, it was possible to identify the contribution of awareness-raising workshops among nursing professionals in the hospital, since there was a change in their knowledge on CHE, perceived by the expansion and deepening of responses both on the CHE concept and on NEPS role, which was manifested in a more coherent way with that presented in scientific literature on the subject. Data convergent with those of this research, in relation to the positive contribution of awareness-raising workshops in knowledge production, were also evidenced in other research carried out in different healthcare contexts^(18,30)^. A study developed by American researchers corroborates this, highlighting the need to plan educational activities for professional development that are integrated into nurses’ professional environments^(31)^.

Thus, the potential of awareness-raising workshops in knowledge production is clear, standing out as a space for learning and production. Furthermore, the knowledge produced from these workshops is characterized as an experience that can foster perceptions and sensitivities that are important for human relationships and, more specifically, for the relationship of care^(13)^.

### Study limitations

Recommendations for conducting qualitative research were followed. However, the fact that data were collected in a single period after the awareness-raising workshop may be a limitation of the findings. It is understood that carrying out follow-up after three and six months of the awareness-raising workshops could contribute to assessing the maintenance of professionals’ knowledge and, if necessary, implementing new awareness-raising workshops on the topic. It is also worth mentioning that CHE is a Brazilian policy, which made it difficult to compare the findings with foreign realities, where the CE concept is more widely used.

### Contributions to nursing and health

This study demonstrates a contribution to nursing and management in hospital healthcare, since it helped in the process of building knowledge about CHE and NEPS, a fact that may lead to changes in the setting, in addition to being a diagnosis for management, enabling reflection on the implementation of NEPS in the institution. Additionally, its contribution to science is clear, as it shows positive results from an intervention made possible by an awareness-raising workshop. This can serve as a model for future research in other contexts with the necessary adaptations.

## FINAL CONSIDERATIONS

This research made it possible to raise awareness among nursing professionals in a hospital about knowledge about CHE. Before the awareness-raising workshop, it was identified that they were unaware or superficial about the topic. Afterwards, it was possible to observe the expansion of knowledge, both in its conceptual and functional aspects. In the conceptual aspect, they were able to describe the meaning and importance of CHE and NEPS in daily life. In the functional aspect, knowledge about CHE as a knowledge driver and NEPS as a tool to put CHE into practice stood out, enabling the personal and professional development of professionals.

Given the above, the contribution of awareness-raising workshops to transforming the knowledge of professionals working in the hospital institution is evident, which can contribute to the care directed at people and to management in terms of thinking about strategies for implementing NEPS in the institution. It is suggested that, based on this initial diagnosis and intervention, future research be conducted with managers and staff of the institution in question, aiming at implementing NEPS. Moreover, research that considers addressing the topic with other professional categories is important.

## Data Availability

The research data are available within the article.

## References

[1] Santos ARD, Santos RMM, Franco TB, Matumoto S, Vilela ABA. (2021). Permanent education in the family health strategy: potentialities and resignifications. Rev Enferm UFPE.

[2] Ribeiro DK, Friedrich DBDC. (2023). Permanent education in health and knowledge management: initiatives in the regional health superintendence. Cogitare Enferm.

[3] Ministério da Saúde (BR) (2021). A Educação Continuada de Enfermeiros do SUS.

[4] Teodosio SS, Nóbrega CBM, Kluczyni CEN, Meneses LBA. (2023). Implantação e desenvolvimento dos núcleos de educação: a percepção dos coordenadores. Saúde Soc.

[5] Santos KCR, Alvarenga LFC. (2022). Núcleo de educação permanente em saúde: relato de experiência. Saberes.

[6] Diniz GC, Diniz MAE, Porcino JMA, Silva MT. (2022). Núcleo de Educação Permanente em Saúde NEPS: ferramenta de gestão do hospital distrital Dr. José Gomes da Silva. Rev Multidiscip Nordeste Min.

[7] Ministério da Saúde (BR) (2004). Portaria nº 198, de 13 de fevereiro de 2004 Institui a Política Nacional de Educação Permanente em Saúde como estratégia do Sistema Único de Saúde para a formação e o desenvolvimento de trabalhadores para o setor e dá outras providências.

[8] Ministério da Saúde (BR) (2007). Portaria nº 1.996, de 20 de agosto de 2007, Dispõe sobre as diretrizes para a implementação da Política Nacional de Educação Permanente em Saúde e dá outras providências.

[9] Carvalho ML, Alcoforado JLM. (2023). Percepção de profissionais sobre a atuação do núcleo de educação permanente em saúde no estado do Maranhão - Brasil. Epistemol Práx Educ.

[10] Lima FJD, Dorneles LL, Pereira MCA, Gatto JR, Góes FDSND, Camargo RAAD. (2022). Permanent health education in a nursing technician course. Rev Esc Enferm USP.

[11] Parente ADN, Ferreira GRON, Cunha CLF, Ramos AMPC, Sá AMM, Haddad MDCFL (2024). Permanent education for quality and patient safety in an accredited hospital. Acta Paul Enferm.

[12] Buljac-Samardzic M, Doekhie KD, van Wijngaarden JDH. (2020). Interventions to improve team effectiveness within health care: a systematic review of the past decade. Hum. Resour. Health.

[13] Finocchioro L, Imbrizi JM. (2017). Oficinas de Arte e a Formação em Saúde: uma experiência no laboratório de sensibilidades da Universidade Federal de São Paulo (Unifesp). Psicol Rev.

[14] Ministério da Saúde (BR), Departamento de Ciência e Tecnologia (2018). Agenda de Prioridades de Pesquisa do Ministério da Saúde - APPMS.

[15] Organização das Nações Unidas no Brasil (2015). A agenda 2030.

[16] Conselho Nacional de Saúde (CNS) (2012). Resolução n° 466. Diretrizes e Normas Regulamentadoras de Pesquisa em Seres Humanos.

[17] Franco MAS. (2005). A pedagogia da pesquisa-ação. Educ Pesqui.

[18] Fernandes F, Casarin F, Greco PBT, Backes DS, Munhoz OL, Ilha S. (2024). Nursing Process for institutionalized older adults: contributions from knowledge awareness workshop. Rev Bras Enferm.

[19] Souza VRDS, Marziale MHP, Silva GTR, Nascimento PL. (2021). Tradução e validação para a língua portuguesa e avaliação do guia COREQ. Acta Paul Enferm.

[20] Moraes R, Galiazzi MC. (2020). Análise textual discursiva.

[21] Bezerra TV, Dias IKR. (2022). Satisfação da enfermagem da atenção primária à saúde com a educação permanente. Rev Baiana Saude Publica.

[22] Backes DS, Bär K, Costenaro RGS, Backes MTS, Souza FGMD, Büscher A. (2022). Permanent education: perception of nursing in the light of complex thought. Acta Paul Enferm.

[23] Hu Y, Wang F, Cao W, Gui C. (2024). Development of training program for the Eye, Ear, Nose, and Throat emergency nurses in China based on core competency: a Delphi study. BMC Emerg Med.

[24] Fontana RT, Thomas LS, Hesler LZ, Guimarães CA. (2021). A educação permanente em saúde na prática de enfermeiras. Rev Contexto Saúde.

[25] Nascimento A, Baduy R. (2021). Simulação, oficina e roda de conversa: estratégias de aprendizagem ativa na saúde. Rev Educ Debate.

[26] Rodrigues GVB, Cortez EA, Almeida YS, Santos ECG. (2021). Processo de educação permanente sob a micropolítica do trabalho vivo em ato de Emerson Merhy: reflexão teórica. Res, Soc Dev.

[27] Moraes MA, Santos AS. (2022). Continuing education: mediation by the management of social assistance work and prevalence of precariousness. Rev Int Econ.

[28] Fakhouri AP, Francischetti I, Vieira CM. (2022). Educação permanente em saúde: concepções e práticas de facilitadores. Rev Interfaces Educ.

[29] Pralon JA, Garcia DC, Iglesias A. (2021). Educação permanente em saúde: uma revisão integrativa de literatura. Res, Soc Dev.

[30] Ilha S, Casarin F, Huppes B, Pires LC, Colpo E, Maziero BR. (2024). Oficinas de sensibilização sobre a doença de Alzheimer no idoso/família: contribuições ao conhecimento em saúde. Rev Kairos.

[31] Dickerson P, Graebe J. (2021). Nursing Continuing Professional Development: a paradigm shift. J Contin Educ. Nurs.

