# Supplementary material for: Raising Awareness about Continuing Health Education with Nursing Professionals: Strategic Action Research
Source: Rev Bras Enferm. 2025 Dec 8;78(5):e20250225. doi: 10.1590/0034-7167-2025-0225 (PMC12707766; doi:10.1590/0034-7167-2025-0225)
Supplement: Translation

# Translation: Sensibilização sobre Educação Permanente em Saúde com Profissionais de Enfermagem: Pesquisa-Ação Estratégica

**DOI:** 10.1590/0034-7167-2025-0225pt

**Authors:** Cloves Roberto Felden da Silva, Juliana Silveira Colomé, Oclaris Lopes Munhoz, Liliane Alves Pereira, Francine Casarin, Silomar Ilha

**Affiliations:** I Universidade Franciscana. Santa Maria, Rio Grande do Sul, Brasil; II Universidade Federal de Santa Maria. Palmeira das Missões, Rio Grande do Sul, Brasil; III Faculdade Integrada de Santa Maria. Santa Maria, Rio Grande do Sul, Brasil

**Keywords:** Educação Continuada, Conhecimento, Hospitais, Pesquisa Qualitativa, Enfermagem.

## Abstract

**Objetivos:**

analisar as contribuições de uma oficina de sensibilização para o conhecimento de profissionais de enfermagem sobre educação permanente em saúde.

**Métodos:**

pesquisa-ação estratégica, desenvolvida com 25 profissionais de enfermagem de hospital privado, situado no Rio Grande do Sul, Brasil. Os dados foram coletados entre janeiro e agosto de 2024, mediante um questionário com questões abertas, antes e após uma oficina de sensibilização. Realizou-se análise textual discursiva dos dados.

**Resultados:**

emergiu a categoria central, “Compreensão acerca da educação permanente em saúde”, unitarizada em cinco categorias de análise que versaram sobre o (des)conhecimento acerca da educação permanente em saúde e sobre o Núcleo de Educação Permanente em Saúde, cenário que mudou após a sensibilização.

**Conclusões:**

revelou-se o desconhecimento ou superficialidade acerca da temática antes das sensibilizações. Após, foi possível identificar a ampliação do conhecimento, tanto no seu aspecto conceitual quanto funcional. Oficinas de sensibilização contribuem para a transformação do conhecimento.

## INTRODUÇÃO

O contexto hospitalar de atenção à saúde é inserido como um dos campos de destaque na atuação dos profissionais de saúde, em especial dos que compõem a equipe de enfermagem, uma vez que estes são os profissionais em maior número neste cenário e, além disso, os que permanecem a maior parte do tempo em contato direto com as rotinas hospitalares e no cuidado. Neste contexto, tornam-se necessários processos de trabalho que permitam aos mesmos o exercício da ação-reflexão-ação nesse cenário de atuação que, por vezes, apresenta rotinas rígidas, preestabelecidas e verticalizadas. Assim, destaca-se a educação permanente em saúde (EPS), com vistas à qualidade do cuidado, pois, com a evolução dos processos de trabalho no Brasil e no mundo, tornou-se fundamental pensar em estratégias que permitam produzir sintonias entre necessidades do campo da saúde e capacidade efetiva de resposta do conjunto dos profissionais^(1)^.

A EPS possui o compromisso com um modo transformador nas práticas de cuidado em saúde, a partir das especificidades territoriais e socioeconômicas^(1)^. Assim, conhecer as potencialidades da EPS nos serviços de saúde configura-se como elemento essencial para superar as adversidades e evidenciar as suas potencialidades^(2)^. A partir da EPS, os profissionais aprendem/fortalecem as práticas mais recentes e baseadas em evidências, o que os capacita/sensibiliza a realizar cuidados que favoreçam a qualidade e segurança dos pacientes, incluindo o uso de novas ferramentas, tecnologias e condutas que podem melhorar os resultados em saúde^(3)^.

Nesse sentido, cientes da necessidade de ações de EPS, instituições de saúde de diferentes regiões do Brasil têm investido esforços na implantação de Núcleos de Educação Permanente em Saúde (NEPS)^(4,5)^. Os NEPS se caracterizam como uma proposta estratégica de gestão, com vistas à consolidação de um serviço organizado para planejar e executar ações de qualificação das práticas em saúde, por meio de processos formativos, com metodologias variadas junto aos profissionais de saúde. A efetivação do NEPS requer um trabalho intersetorial capaz de articular o desenvolvimento individual e institucional^(6)^.

Embora a implantação do NEPS não conste como diretriz da Política Nacional de Educação Permanente em Saúde (PNEPS)^(7)^ e da Portaria GM/MS nº 1.996/2007, que dispõe sobre as diretrizes para a implementação da PNEPS^(8)^, o mesmo tem se demonstrado como uma importante ferramenta de consolidação da EPS em diferentes cenários. Nesse sentido, pesquisa realizada no estado da Paraíba demonstrou que a implantação do NEPS contribuiu para o trabalho em colaboração, o que potencializou a viabilização de ações para fortalecer a EPS. Além disso, a pesquisa evidenciou que o NEPS contribuiu para o interesse dos coordenadores em utilizar diferentes abordagens de metodologias ativas de trabalho junto aos colaboradores^(4)^. Outra pesquisa realizada no estado do Maranhão demonstrou que a implementação do NEPS foi fundamental, por sua característica de transformação, melhora da qualidade da assistência, e promoção de cuidados qualificados, responsáveis e conscientes^(9)^.

Os NEPS possuem a finalidade de elaborar, planejar, apoiar e executar, de modo articulado, as propostas que envolvem ações educativas no trabalho, de forma que o aprender e o ensinar se incorporam no cotidiano dos serviços e no trabalho em saúde^(10)^ Dessa forma, nas instituições que implantaram o NEPS, as atividades de EPS são estratégias desenvolvidas, principalmente, pelas ações do mesmo, com objetivo de melhorar os processos de formação dos trabalhadores, reorganização das práticas em saúde, a partir de um arcabouço teórico de ensino-aprendizagem^(4-9)^.

Contudo, embora compreendam-se a relevância e necessidade do NEPS nos serviços de saúde, ainda é possível perceber que não é uma realidade em todas as instituições, como no cenário desta pesquisa, o que requer atenção e investimento para sua efetivação, manutenção ou melhoria da qualidade dos serviços de atenção à saúde. Dessa maneira, torna-se necessário compreender o que os profissionais de saúde dessas instituições sabem sobre a EPS e sobre o NEPS como ferramenta potencializadora das ações e EPS, com vistas ao diagnóstico situacional que possibilite pensar em estratégias para sua futura implementação^(11)^.

Além disso, entende-se que, para a efetivação de uma proposta de trabalho com a viabilização da implementação de um NEPS no cenário da pesquisa, é fundamental que os profissionais compreendam a importância da EPS e do NEPS como ferramentas potencializadoras de ações educativas/formativas. Para tanto, é crucial investir em metodologias e estratégias que contribuam para o conhecimento dos profissionais acerca do assunto. Nos países desenvolvidos, o investimento em atividades que proporcionam a capacitação de profissionais de saúde tem sido considerada uma estratégia de intervenção amplamente utilizada^(12)^.

Entre as metodologias possíveis para esse fim, destacam-se as oficinas de sensibilização/capacitação, pois oportunizam a instrumentalização dos participantes, por meio de metodologias que valorizam a construção participativa e a troca de experiências e de conhecimentos^(13)^. Em pesquisa realizada com profissionais de um hospital no estado do Pará, os gestores relataram os desafios no processo de EPS, destacando a necessidade de dedicar esforços com vistas à sensibilização dos profissionais. Da mesma forma, os profissionais de saúde reconhecem a importância e necessidade de diferentes metodologias para a sensibilização/capacitação, como a utilização de oficinas^(11)^.

Contudo, para além de realizar ações de intervenção, torna-se necessário avaliar a contribuição/efetividade dessas, por meio da produção e/ou aprofundamento do conhecimento, fato que justifica a necessidade e relevância desta pesquisa que, além de oferecer oficinas de sensibilização/capacitação, propõe-se a comparar o conhecimento dos profissionais antes e após as mesmas, o que pode contribuir para a gestão hospitalar, para a efetivação do processo de melhoria contínua da qualidade. Além do até aqui descrito, salienta-se que as discussões e pesquisas sobre a temática em tela são essenciais e necessárias, uma vez que a mesma é destacada pela Agenda Nacional de Prioridades de Pesquisa em Saúde no país, mais especificamente no eixo 8 (gestão do trabalho e educação em saúde)^(14)^. Também, encontra-se alinhada ao objetivo 3 (“Saúde e bem-estar”) e objetivo 8 (“Trabalho decente e crescimento econômico”) para o desenvolvimento sustentável, apresentado na Agenda 2030 da Organização das Nações Unidas^(15)^. Diante do exposto, questiona-se: quais as contribuições de uma oficina de sensibilização para o conhecimento de profissionais de enfermagem sobre EPS?

## OBJETIVOS

Analisar as contribuições de uma oficina de sensibilização para o conhecimento de profissionais de enfermagem sobre EPS.

## MÉTODOS

### Aspectos éticos

Esta pesquisa faz parte de um macroprojeto denominado “Implementação do Núcleo de Educação Permanente em Saúde em um hospital privado: pesquisa ação estratégica”. O referido projeto teve o objetivo geral de construir o NEPS em hospital privado. Este artigo contempla um dos objetivos específicos do projeto.

O presente estudo foi submetido à apreciação de um Comitê de Ética em Pesquisa com Seres Humanos (CEP) via Plataforma Brasil. Somente após a aprovação do CEP, realizou-se o primeiro contato com os participantes. Consideraram-se os preceitos éticos e legais que envolviam a pesquisa com seres humanos, conforme a Resolução nº 466/12 do Ministério da Saúde^(16)^. O Termo de Consentimento Livre e Esclarecido foi assinado pelos participantes em duas vias; uma ficou com ele e a outra com os pesquisadores. Manteve-se o anonimato dos participantes, identificando-os pelas letras “E” (enfermeiro) e “TE” (técnico de enfermagem), seguida de um algarismo (E1, E2...; TE1, TE2...).

### Tipo de estudo

Trata-se de pesquisa-ação estratégica^(17)^, cuja transformação foi previamente planejada pelo pesquisador, o qual foi responsável por acompanhar os efeitos e avaliar os resultados de sua aplicação. Para tanto, como condução desta pesquisa-ação estratégica, foram seguidas quatro fases, dividida em oito passos, conforme pode ser visualizado no Quadro 1.

**Quadro 1:** Representação esquemática das fases da pesquisa-ação estratégica

PESQUISA-AÇÃO		
**Fase**	**Passos da pesquisa**	
Diagnóstica	Exploratória	Identificação do problema dentro de um contexto Levantamento dos dados pertinentes
	Analítica	Análise dos dados levantados Significado dos dados levantados Identificação das necessidades de mudança
Planejamento	Encontrando possíveis soluções	
Implementação	Intervenção ação	
Avaliação	Transformação	

*Fonte: retirado de^(18)^.*

Para nortear a clareza e redação deste relatório, utilizou-se o *checklist COnsolidated criteria for REporting Qualitative research* ^(19)^.

### Procedimento metodológicos

#### Cenário do estudo

Pesquisa desenvolvida em hospital privado, localizado na região central do estado do Rio Grande do Sul, Brasil. A referida instituição foi fundada em 1976. Atualmente, possui 68 leitos de internação, incluindo unidades de internação clínica, cirúrgica, psiquiatria e pós-cesariana. Também possui cinco salas de cirurgias, dez leitos na sala de recuperação e cinco leitos de Unidade de Terapia Intensiva, com a finalidade de proporcionar um cuidado adequado, mantendo-se com recursos de seu trabalho particular e convênios.

A instituição possui 200 profissionais, sendo 25 enfermeiros, 100 técnicos de enfermagem, uma nutricionista, quatro fisioterapeutas, uma psicóloga, um administrador, 22 profissionais responsáveis pela higienização, cozinheiras, profissionais de serviços gerais, além de médicos clínicos e cirurgiões.

#### Participantes do estudo

Embora compreenda-se que a EPS opera pela lógica interprofissional e que, em um cenário de serviço de saúde, todos cuidam, para esta pesquisa, optou-se por trabalhar no primeiro momento com os profissionais da equipe assistencial de enfermagem da instituição, por serem os que atuam diretamente nos cuidados diretos aos pacientes, desde o momento de internação até a alta hospitalar. Além disso, por serem os que normalmente realizam a gestão (enfermeiros) e organização (equipe de enfermagem) dos serviços de saúde, orientam outros profissionais acerca das atualizações de normas e rotinas institucionais.

Foram incluídos enfermeiros ou técnicos de enfermagem atuantes na referida instituição a, no mínimo, três meses, pois compreende-se que os profissionais já passaram pelo período de experiência e fazem parte do quadro efetivo de colaboradores da instituição. Foram considerados excluídos do estudo os profissionais que estiveram de atestado médico, licença ou qualquer outro afastamento no período de coleta de dados. Atenderam aos critérios e aceitaram participar da pesquisa 25 profissionais.

#### Coleta e organização dos dados

Os dados foram coletados de 25 participantes em dois momentos. Inicialmente, de janeiro a março de 2024, eles foram convidados individualmente, pelo pesquisador principal, a participar da pesquisa. Após o aceite, foi realizada, entre junho e julho de 2024, a entrevista semiestruturada (momento 1), construída especificamente para esta pesquisa, sendo realizada em um único momento com cada participante. O roteiro de entrevista foi composto de duas partes: a primeira, com a descrição dos participantes, e a segunda, com as seguintes questões abertas: o que você compreende por EPS? O que você compreende por NEPS?

A coleta dos dados foi conduzida por um dos pesquisadores, enfermeiro, do sexo masculino, com experiência no contexto hospitalar, o qual foi previamente capacitado para a coleta dos dados. Realizou-se teste piloto do instrumento, com a aplicação da entrevista com um enfermeiro e um técnico de enfermagem, que não compuseram a amostra desta pesquisa. As entrevistas (momento 1) permitiram verificar lacunas do conhecimento acerca da temática, o que conduziu à necessidade da intervenção. Assim, os pesquisadores buscaram encontrar possíveis soluções e, como intervenção, planejaram oficinas com vistas à sensibilização dos profissionais de enfermagem sobre a importância da EPS e do NEPS, que ocorreram nos meses de julho e agosto de 2024.

Foram realizadas três oficinas nas unidades do hospital, com duração média de 40 minutos cada uma delas. A atividade foi coordenada pelo pesquisador, enfermeiro, do sexo masculino, com experiência nas temáticas abordadas. Iniciaram-se as oficinas com as boas-vindas aos participantes e agradecendo pela participação na pesquisa. Como recurso visual, utilizou-se uma apresentação realizada no *PowerPoint* com aspectos relacionados à EPS e ao NEPS. Os profissionais foram estimulados a contribuir com as oficinas, compartilhando os seus conhecimentos e suas experiências. Após esse momento inicial, realizou-se uma discussão coletiva sobre as temáticas, proporcionando ampliar e aprofundar o conhecimento sobre as referidas temáticas.

Após as oficinas de sensibilização, de agosto a setembro de 2024, foram realizadas novamente as entrevistas semiestruturadas (momento 2) com os 25 participantes, utilizando-se o mesmo instrumento empregado no primeiro momento, com duração média de 40 minutos, com vistas à avaliação do conhecimento acerca da temática, após a intervenção. Salienta-se que todas as entrevistas foram, com a autorização dos participantes, registradas manualmente. Na sequência, foram transcritas na íntegra mecanograficamente pelos pesquisadores, com o auxílio do programa *Microsoft Word* ^®^ (versão 16.31). As mesmas foram devolvidas aos participantes, para que eles validassem as informações.

#### Análise dos dados

Os dados foram analisados com base na técnica da análise textual discursiva, a partir dos seus três componentes: unitarização; estabelecimento de relações; e comunicação^(20)^. Assim, os pesquisadores iniciaram pela leitura dos textos de forma intensa e aprofundada, o que conduziu a uma categoria central acerca da compreensão dos profissionais sobre a EPS. Após, os relatos inicialmente inseridos nessa categoria foram lidos novamente e de forma minuciosa, sendo separados em diferentes unidades de sentido, das quais se originaram cinco categorias (Figura 1). Por fim, os pesquisadores realizaram o processo de comunicação, momento em que produziram as descrições e interpretações dos fenômenos investigados^(20)^.

**Figura 1:** Esquema representativo da interligação entre a categoria central e as categorias de análise

## RESULTADOS

Dos 25 participantes da pesquisa, 21 eram mulheres e quatro eram homens, com idades entre 24 e 53 anos. Quanto à formação, 15 eram técnicos de enfermagem e nove eram enfermeiros. No que se refere ao tempo de formação, variou de dois a 22 anos. O tempo de experiência profissional variou de oito meses a 21 anos, e a atuação no hospital cenário da pesquisa variou de oito meses a 16 anos.

Os dados analisados resultaram em uma categoria central intitulada “Compreensão acerca da educação permanente em saúde” e em cinco categorias de análise, conforme a Figura 1.

### Educação permanente em saúde: conhecimento pontual e linear

Antes das oficinas de sensibilização, os técnicos de enfermagem apresentavam conhecimento pontual e linear sobre a temática, uma vez que suas falas versaram apenas sobre capacitações e treinamentos, o que não caracteriza em completude o conceito de EPS. Os enfermeiros, ainda que tenham demonstrado compreenderem melhor o assunto, apresentaram falas ainda pouco contemplativas a definição e objetivos da EPS.

*Para mim, significa aprender com capacitações.* (TE2)

*Trabalho com o objetivo de uma continuação de assistência voltada para os usuários.* (TE4)

*Acredito que seja treinamentos e capacitações para manter o aprendizado dos profissionais na rotina do trabalho.* (TE5)

*Manter atualizado o profissional dentro de uma instituição.* (TE10)

*Estratégias para mudanças na saúde*. (TE13)

*Treinamentos nas unidades mensal ou quinzenal*. (TE14)

[...] *uma educação contínua sobre um determinado assunto do nosso serviço*. (E1)

*Tem a finalidade educacional, responsável pelas capacitações profissionais.* (E2)

*Compreendo com treinamentos a serem desenvolvidos para profissionais atuantes em um determinado serviço.* (E4)

*São ações dentro do serviço que buscam qualificar a prática profissional.* (E5)

*Criar ações educativas com algum tema de problematização no trabalho*. (E7)

*É uma articulação das práticas observando os problemas e fraquezas dos setores e com isso transformar essas necessidades em treinamentos.* (E9)

### Núcleo de Educação Permanente em Saúde: (des)conhecimento sobre o conceito e a função

No que se refere ao conhecimento dos profissionais acerca do NEPS, antes das oficinas de sensibilização, alguns participantes fizeram referência à EPS. Outros profissionais se referiram ao NEPS como um setor para realização de treinamentos e capacitações.

*Seria uma educação continuada para os profissionais da saúde.* (TE1)

*Um setor que dá treinamentos para os funcionários,* (TE3)

*Acredito que o intuito seja o atendimento de forma mais eficaz.* (TE6)

*Seria um setor com alguém para realizar os treinamentos para os funcionários.* (TE7)

*Acho que seja um setor que dê treinamentos.* (TE9)

*Acho que serve para trazer novas técnicas de trabalho.* (TE11)

*Serve para melhorar a capacitação profissional dos funcionários.* (TE15)

*Qualificação e capacitação profissional.* (E3)

*Compreendo como setor responsável por capacitar as equipes atuantes de um hospital.* (E6)

No que diz respeito à função do NEPS, antes das oficinas de sensibilização, os participantes referiram melhorar os atendimentos, as técnicas de trabalho, capacitações e educação continuada (EC).

### Presença de treinamentos e capacitações: ausência do Núcleo de Educação Permanente em Saúde

Conforme o relato dos participantes, é possível perceber que existem treinamentos em alguns setores do hospital, os quais são passados pelos enfermeiros das unidades às equipes. Contudo, os participantes da pesquisa desconheciam a presença de um NEPS na instituição.

*Não, pois temos apenas treinamentos que na maioria das vezes já são do conhecimento do profissional, porém, muitas vezes, não é exercido com frequência*. (TE8)

*Falta alguém que abrace a causa para ser feito. Falta pessoas para integrar o núcleo que visaria melhorias, talvez por esse motivo ainda não exista. Também aqui no hospital tem muita rotatividade de enfermeiros.* (TE9)

*Ainda não temos um NEPS; temos apenas os treinamentos realizados pelos enfermeiros das unidades. Falta organização e planejamento da instituição.* (TE10)

*Não, por conta de fragilidades no desenvolvimento, faltam pessoas para integrar um núcleo. Aqui no hospital tem muita rotatividade de enfermeiros.* (TE12)

*Não, acredito que por não saberem sua real importância e falta de planejamento* [...] *porque precisa de investimento para contratação de um profissional responsável para chefiar esse núcleo, talvez falte iniciativa de alguém, não sei.* (E3)

*Não, não existe por falta de organização e criação, pois, na minha opinião, falta interesse por parte dos administradores e gerência.* (E5)

*Não, sinceramente não sei informar porque não existe ainda; acredito que a rotatividade de profissionais impede que seja criado o núcleo de educação permanente.* (E8)

*Não, não existe o núcleo, porém existem treinamentos dentro da instituição.* (E9)

Nas suas percepções, faltam profissionais capacitados, organizados, dedicados e com planejamento adequado que estejam disponíveis para integrar e se responsabilizar. Os profissionais atribuem a não efetivação do NEPS, entre outros fatores, à falta de organização, de investimento, de profissionais para chefiar os serviços e da alta rotatividade de profissionais na instituição.

### Educação permanente em saúde: conhecimento ampliado e contextualizado

Após a realização da oficina de sensibilização, notaram-se avanços no conhecimento dos profissionais sobre a EPS. Os participantes demonstraram uma compreensão mais ampla em relação à contribuição da EPS como propulsora de conhecimento.

*São ações que aprimoram os conhecimentos relacionados ao trabalho, compartilhamento de saberes com momentos de escuta e de fala, abordando assuntos que incentivem a promoção e a prevenção em saúde por meio de intervenções educativas.* (TE2)

*Trata-se de toda atividade educativa que visa à problematização da assistência prestada, além de servir como um propulsor do conhecimento por meio das experiências adquiridas e compartilhadas. É uma ferramenta para refletir e compartilhar experiências, visando um trabalho mais qualificado, promovendo o pensamento crítico, reflexivo, compromissado e tecnicamente qualificado e aprofundando determinados assuntos que visam à demanda para as equipes, melhorando a qualidade do serviço.* (TE5)

*Consiste em ações educativas e refere-se à necessidade de saúde das pessoas e das populações. Aprofunda determinados assuntos que surgem a partir das demandas das equipes. Visa melhorar a qualidade dos serviços. É uma ferramenta para refletir e compartilhar experiências, visando um trabalho mais qualificado, transformando o ambiente de trabalho. Tratam-se de ações idealizadas junto com as equipes, ouvindo os profissionais e compreendendo as suas necessidades e, assim, construindo condições para qualificação desses profissionais e melhorar o atendimento para os pacientes.* (TE10)

*São ações educativas embasadas na problematização do processo de trabalho em saúde. Uma ferramenta para refletir e compartilhar experiências, visando um trabalho qualificado, transformar o ambiente de trabalho, promovendo o pensamento crítico e reflexivo. O prender e o ensinar devem se incorporar ao cotidiano das organizações e ao trabalho.* (E4)

*Entendo como uma forma de observar e perguntar as necessidades dos profissionais e promover ações diversas, buscando melhorar a qualidade e efetividade do trabalho.* (E5)

*É quando se traz práticas cotidianas e organiza-se um ensino, um método de aprendizagem, onde todos da instituição de saúde podem aprender e assim melhorar seus atos laborais, transformando, assim, esses atos de forma mais eficaz, qualificada e segura.* (E9)

Como pode-se perceber, após as oficinas de sensibilização, os participantes da pesquisa passaram a compreender a EPS como uma ferramenta que possibilita momentos de escuta e de diálogos, com oportunidades de aprendizado, construção de conhecimento enquanto profissional e melhorias na qualidade do serviço. Além disso, os relatos a seguir demonstram a compreensão dos participantes acerca das funções da EPS.

*Promove o pensamento crítico, reflexivo e compromissado e tecnicamente qualificado, e aprofundando determinados assuntos que visam à demanda para as equipes, melhorando a qualidade do serviço.* (TE4)

*Tem o intuito de melhorar os serviços de saúde, também o compartilhamento de saberes com momentos de escuta e de fala, onde temos a oportunidade de aprender, desaprender e reconstruir enquanto profissional.* (TE13)

*Na minha opinião, a educação permanente oportuniza o aprender com o cotidiano, compartilhamento de saberes, momentos de escuta, de aprendizado, incentivar a promoção da saúde e a prevenção de agravos por meio de atividades educativas*. (TE14)

*Serve para qualificar o trabalhador, atualizar as rotinas e busca constante de conhecimento, incentiva a promoção e a prevenção em saúde por meio de intervenções educativas com compartilhamento de saberes e momentos de escuta e de fala*. (E2)

*A educação permanente objetiva a transformação do conhecimento que se reflete no serviço, aprimoramento, atualização dos serviços, visando à qualidade na ampliação dos conhecimentos. Por isso, compreendo como uma ferramenta para refletir e compartilhar experiências, visando à qualidade no e do trabalho* [...]. (E1)

*Aprendizagem no trabalho, onde o aprender e o ensinar estão associados ao cotidiano. Tem por finalidade a identificação das necessidades dos profissionais e do serviço e, com isso, buscar a possibilidade de transformar e melhorar atuação dos trabalhadores da saúde.* (E7)

### Núcleo de Educação Permanente em Saúde: ferramenta propulsora de desenvolvimento pessoal e profissional

Após as oficinas, pode-se perceber que os profissionais passaram a compreender o NEPS como propulsor de crescimento pessoal e profissional, em que o aprendizado e o processo educativo caminham juntos aprimorando novas técnicas de trabalho e outras necessidades que surgem no cotidiano de trabalho.

*Um grupo formado por pessoas capacitadas com o objetivo de proporcionar a propulsão de conhecimentos, de desenvolvimento pessoal e profissional dos profissionais.* (TE1)

*Tem por objetivo o desenvolvimento pessoal e profissional dos funcionários através de novos conhecimentos, técnicas e processos de trabalho, capacitando-os para o melhor cuidado e colaboração com a equipe.* (TE3)

*O NEPES contribui para o desenvolvimento pessoal e profissional dos profissionais; tem o objetivo de apresentar novos conhecimentos para melhorias o trabalho, melhoria na qualidade dos serviços de atenção à saúde.* (TE6)

*Tem por objetivo proporcionar o desenvolvimento pessoal e profissional dos funcionários, através de novos conhecimentos, técnicas e processo de trabalho*. (TE7)

*Um grupo de profissionais responsáveis por desenvolver o crescimento técnico, pessoal e profissional dos colaboradores de uma determinada instituição*. (TE9)

*Seria um grupo de profissionais responsáveis por desenvolver o crescimento técnico, pessoal e profissional dos colaboradores de uma determinada instituição*. (TE11)

*Proporcionar o desenvolvimento pessoal e profissional dos funcionários, através de conhecimentos, instrumentalizando-os para o cuidado em equipe multiprofissional*. (TE15)

*Atua na capacitação da equipe multiprofissional para melhorar a qualidade dos serviços através de novos conhecimentos, técnicas e processos de trabalho, capacitando-os para a execução adequada de melhores cuidados em saúde e atuação com a equipe multiprofissional, garantindo um atendimento de qualidade*. (E3)

*É um núcleo formado por pessoas que buscam propiciar o desenvolvimento pessoal e profissional dos colaboradores, através de capacitações para que o cuidado seja adequado com técnicas e processos de trabalho adequadas e seguros para a realidade.* (E6)

## DISCUSSÃO

Na presente pesquisa, evidenciou-se que, antes da oficina de sensibilização, parte dos participantes, especialmente os técnicos de enfermagem, apresentava desconhecimento sobre a EPS, uma vez que suas falas versaram predominantemente sob elementos como capacitações e treinamentos, o que não conversa com a amplitude do conceito e função da EPS. Dado semelhante foi evidenciado em pesquisa realizada em hospital de grande porte, na cidade de Curitiba, no estado do Paraná, onde os participantes se referiram à EPS como sinônimo de EC, não possuindo uma visão aprofundada sobre o tema e a distinção sobre dos conceitos^(21)^.

A esse respeito, cabe mencionar que a EPS objetiva mais do que a realização de capacitações e treinamentos, pois representa uma cultura de educação no serviço propulsora de desenvolvimento pessoal e profissional das pessoas envolvidas, em que a observação, o diálogo e a escuta ativa devem estar constantemente presentes para que as necessidades das pessoas envolvidas possam ser identificadas. Entende-se que, a partir disso, seja possível pensar e planejar ações que contribuam com a realidade, possibilitando a autorreflexão e autoavaliação, contribuindo para que cada profissional se sinta o autor/protagonista no percurso de sua aprendizagem ao longo da vida^(22)^.

Assim, denota-se que a EPS é um conceito pedagógico que deve ser definido como aprendizagem no ambiente de trabalho, onde o ensinar e o aprender se formam no cotidiano das organizações e no trabalho. Está atrelada na aprendizagem significativa e na possibilidade de transformar as práticas profissionais e o ambiente de trabalho, ao englobar diversos alicerces entre a realidade e experiência de cada pessoa, solicitando o encontro de uma solução para a problemática enfatizada. Estudo desenvolvido na China destacou a importância de programas de educação permanente adaptados às necessidades específicas para melhorar a competência profissional dos enfermeiros^(23)^. Dessa forma, pode-se inferir que a EPS se caracteriza como uma ferramenta que proporciona aos profissionais uma proximidade da teoria e prática, permitindo soluções e mudanças do cenário e da assistência prestada à comunidade^(24)^.

No que se refere ao conhecimento sobre o NEPS, antes das oficinas de sensibilização, os profissionais participantes da pesquisa o compreendiam como um setor para realização de treinamentos e capacitações de novas técnicas, assim como EC. Esse dado corrobora os de pesquisa desenvolvida com o objetivo de implementar o NEPS em hospital psiquiátrico no estado do Piauí, a qual demonstrou dado semelhante, uma vez que foram atribuídas ao mesmo as funções de corrigir fragilidades e aperfeiçoar as práticas dos trabalhadores^(25)^.

Como pode-se perceber, os participantes da presente pesquisa trazem nas suas falas uma compreensão limitada e simplista do NEPS, atribuindo, ao mesmo, apenas a função de capacitação e treinamentos. Assim, é importante mencionar que planejar e realizar capacitações e treinamentos no serviço são algumas das funções do NEPS; contudo, a sua relevância nos serviços não se restringe apenas à execução de tarefas pontuais. O NEPS é a estrutura responsável pelo trabalho com a realidade dos serviços, definindo uma conexão entre educação e trabalho, de forma a retomar o processo de EPS para o desenvolvimento dos serviços e geração de impacto na saúde do usuário^(26)^.

Nesse sentido, cabe mencionar que o desconhecimento das questões conceituais e operacionais da EPS e do NEPS, assim como entre a EPS e EC, provoca a reflexão quanto à necessidade de ampliar a discussão para a formação acadêmica e técnica, que não possibilitou aos profissionais, participantes da presente pesquisa, conhecerem a PNEPS. Além disso, outro fator relevante a ser questionado é a ausência de espaços de problematização no cotidiano do serviço.

A partir dos relatos, foi possível identificar que existiam treinamentos em alguns setores do hospital cenário da presente pesquisa, os quais eram passados pelos enfermeiros das unidades. Contudo, não existia o NEPS implementado na instituição em decorrência, conforme os participantes, da falta de organização, de investimento, de profissionais para chefiar os serviços e da alta rotatividade de profissionais na instituição. Dado semelhante foi evidenciado em uma pesquisa desenvolvida em um hospital público, com o objetivo de compreender os desafios enfrentados pela EPS para o alcance da melhoria da qualidade e da segurança do paciente. Na pesquisa em questão, os desafios versaram desde questões humanas, como a resistência à mudança; questões gerenciais e a alta rotatividade de profissionais na instituição^(11)^.

A esse respeito, pesquisa desenvolvida em um hospital na cidade de Belém, no estado do Pará destacou a importância da EPS para a melhoria da qualidade. A EPS foi apontada como a base desse processo, onde os profissionais se mantem constantemente atualizados em relação aos protocolos de segurança do paciente e à execução dos procedimentos adequados a serem realizados, estimulando na equipe multidisciplinar, a aquisição de conhecimentos, habilidades e atitudes, que contribuem para o gerenciamento de riscos, a garantia da qualidade e a segurança nos processos assistenciais^(27)^.

Nesse sentido, cabe mencionar que o NEPS nos serviços de saúde se constitui como ferramenta que permite aos profissionais já incluídos no serviço constante atualização dos conhecimentos e integração das disciplinas em diferentes saberes em saúde. Além disso, possui rotinas e cronogramas de reuniões com os representantes da gestão, sendo responsável pelo levantamento das necessidades de aprendizagem dos trabalhadores das unidades de saúde e pelo desenvolvimento de ações de educação em saúde^(28)^.

Diante disso, as ações de EPS são oportunidades para produção de conhecimentos e maior segurança, contribuindo para os processos de trabalho entre os profissionais, fortalecendo, dessa forma, o trabalho em equipe, pois o ato de planejar exige conhecimento da realidade que permitam traçar objetivo e metas. Dessa forma, é importante que a equipe do NEPS atue conjuntamente com o sistema organizacional da instituição, para garantir sua atuação apesar das mudanças existentes^(29)^.

Nesse sentido, destaca-se a necessidade de superar a resistência, por meio da sensibilização dos profissionais de saúde quanto à importância dos processos educativos^(27)^. Na presente pesquisa, após as oficinas de sensibilização, os profissionais passaram a compreender melhor os conceitos e atribuições/contribuições da EPS como propulsora de crescimento técnico, pessoal e profissional dos trabalhadores, o que repercute no cuidado de qualidade, eficiente e seguro aos pacientes. O mesmo ocorreu em relação ao NEPS, uma vez que os profissionais passaram a compreendê-lo para além de um setor, como uma ferramenta que oportuniza colocar em prática a EPS e seus benefícios, por meio da escuta, diálogos, aprendizado e construção de conhecimento, repercutindo na qualidade do serviço.

Dessa forma, foi possível identificar a contribuição das oficinas de sensibilização junto aos profissionais de enfermagem no contexto hospitalar, uma vez que ocorreu a mudança de conhecimento destes sobre a temática da EPS, percebida pela ampliação e aprofundamento das respostas tanto sobre o conceito de EPS quanto sobre a função do NEPS, a qual se manifestou de forma mais coerente com que apresenta a literatura científica sobre a temática. Dados convergentes aos da presente pesquisa, em relação à contribuição positiva de oficinas de sensibilização na produção de conhecimento, também foram evidenciados em outras pesquisas realizadas em diferentes contextos de atenção à saúde^(18,30)^. Corroborando, estudo desenvolvido por pesquisadores americanos destacou a necessidade do planejamento de atividades educativas de desenvolvimento profissional integrado ao ambiente profissional dos enfermeiros^(31)^.

Assim, percebe-se o potencial das oficinas de sensibilização na produção de conhecimento, destacando-se como um espaço de aprendizagem e produção. Ademais, o conhecimento produzido a partir dessas caracteriza-se como uma experiência que pode favorecer percepções e sensibilidades importantes para as relações humanas e, mais especificamente, para a relação de cuidado^(13)^.

### Limitações do estudo

Foram seguidas as recomendações para a condução de pesquisas qualitativas. Contudo, a realização a partir da coleta dos dados em um único período após a oficina de sensibilização pode ser uma limitação dos achados. Entende-se que a realização do *follow-up* após três e seis meses das oficinas de sensibilização poderia contribuir para avaliação da manutenção do conhecimento dos profissionais e, se necessário, implementação de novas oficinas de sensibilização sobre a temática. Cabe mencionar também que a EPS é uma política brasileira, o que dificultou a comparação dos achados com realidades estrangeiras, onde o conceito de EC é mais empregado.

### Contribuições para as áreas da enfermagem e saúde

Este estudo demonstra contribuição para a enfermagem e gestão no contexto de atenção à saúde hospitalar, uma vez que auxiliou no processo de construção de conhecimentos sobre a EPS e sobre o NEPS, fato que poderá repercutir em mudança no cenário, além de ser um diagnóstico à gestão, possibilitando refletir na implementação do NEPS na instituição. Somado a isso, compreende-se sua contribuição para a ciência, pois demonstra o resultado positivo após uma intervenção possibilitada a partir de uma oficina de sensibilização, a qual poderá servir de modelo para pesquisas futuras em outras realidades, com as devidas adaptações necessárias para cada contexto/realidade.

## CONSIDERAÇÕES FINAIS

Esta pesquisa possibilitou sensibilizar os profissionais de enfermagem de um hospital sobre o conhecimento acerca da EPS. Antes da oficina de sensibilização, identificou-se o desconhecimento ou superficialidade acerca da temática. Após, foi possível constatar a ampliação do conhecimento, tanto no seu aspecto conceitual quanto funcional. No aspecto conceitual, souberam descrever o significado e importância da EPS e do NEPS no cotidiano. No aspecto funcional, destacou-se o conhecimento sobre a EPS propulsora de conhecimento e o NEPS como ferramenta para colocar em prática a EPS, possibilitando o desenvolvimento pessoal e profissional dos profissionais.

Diante do exposto, denota-se a contribuição das oficinas de sensibilização para a transformação do conhecimento dos profissionais atuantes na instituição hospitalar, o que pode contribuir com o cuidado direcionado às pessoas e com a gestão no que se refere a pensar em estratégias para a implementação do NEPS na instituição. Sugere-se que, a partir desse diagnóstico inicial e intervenção realizados, pesquisa futura seja conduzida com os gestores e colaboradores da referida instituição, visando à implementação do NEPS. Somado a isso, pesquisas que considerem abordar a temática com outras categorias profissionais são importantes.

## Data Availability

Os dados da pesquisa estão disponíveis no corpo do artigo.
